# The Inverse Relationship between Influenza Vaccination and Antimicrobial Resistance: An Ecological Analysis of Italian Data

**DOI:** 10.3390/vaccines10040554

**Published:** 2022-04-03

**Authors:** Martina Barchitta, Andrea Maugeri, Rosario Vinci, Antonella Agodi

**Affiliations:** Department of Medical and Surgical Sciences and Advanced Technologies “GF Ingrassia”, University of Catania, Via S. Sofia 87, 95123 Catania, Italy; martina.barchitta@unict.it (M.B.); andrea.maugeri@unict.it (A.M.); rosario.vinci@studium.unict.it (R.V.)

**Keywords:** vaccines, antibiotic, AMR, public health, drug resistance

## Abstract

Antimicrobial resistance (AMR) is one of the key threats to global health and requires sustainable and effective actions to reduce its consequences in the near future worldwide. There are several well-documented and epidemiologically supported examples that both bacterial and viral vaccines might have an impact on AMR. Here, we conducted an ecological analysis of Italian data to evaluate the relationship between influenza vaccination coverage and AMR proportions over the last two decades. We found significant negative correlations between influenza vaccination coverage in the population over 64 years and AMR in *Escherichia coli* and *Klebsiella pneumoniae* isolates. The observed inverse relationships were confirmed by linear regression analysis. Similar results were obtained for the vaccination coverage in the overall population. Despite the main limitations of our study, its findings suggested an inverse relationship between influenza vaccination coverage and AMR proportions over the years, which was in accordance with previous theories illustrating how influenza vaccination reduced antibiotic prescriptions. However, since our study did not allow for us to elucidate the mechanisms underpinning the observed relationship, more and better data on confounding and mediating factors should be considered in future research.

## 1. Introduction

Antimicrobial resistance (AMR) is one of the most important challenges that modern medicine must face. Each year, there are over 670,000 infections due to antimicrobial-resistant bacteria in the European Union (EU/EEA) and approximately 33,000 deaths as a direct result of these infections [1]. It has been estimated that the number of deaths due to AMR will exceed those due to cancer by 2050 [2]. To date, the burden of AMR is comparable to that of influenza, tuberculosis, and Human Immunodeficiency Virus-Acquired Immune Deficiency Syndrome (HIV-AIDS) combined [3]. A recent systematic analysis estimated that, in 2019, about 4.95 million deaths were associated with AMR worldwide, including those caused directly by AMR. These estimates corresponded to an annual burden of nearly 50,000 Disability Adjusted Life Years (DALYs) [4]. Although assessing the exact burden of AMR remains difficult in many settings, the occurrence of specific AMR microorganisms is evident in several healthcare settings covered by surveillance systems [3]. A major issue regards the resistance to last-line antimicrobials (e.g., vancomycin and carbapenems). In particular, high percentages of *Klebsiella pneumoniae* resistant to third-generation cephalosporins and carbapenems have been reported in several countries [3]. Similarly, high percentages of *Acinetobacter spp.* that are resistant to carbapenems are of concern [3]. This situation is mostly ascribed to serious limitations in treatment options for infections associated with these microorganisms, which cause the spread of these resistant clones in healthcare setting and in the community, at both national and international levels. AMR pathogens, in fact, cannot be contained within borders, raising the need for joint actions to contrast AMR throughout the World Health Organization (WHO) regions.

Thus, robust investments to tackle AMR are urgently necessary and would have a significant positive effect on healthcare systems, as well as on population health in general. For these reasons, efforts to prevent and control AMR are essential for the Public Health agenda. As AMR is among the ten key threats to global health [5], the heads of the Food and Agriculture Organization of the United Nations (FAO), the World Organization for Animal Health (OIE), and the WHO launched the new One Health Global Leaders Group on AMR. This group is committed to catalyzing global attention and actions to preserve antimicrobial drugs and avert the disastrous consequences of AMR [6]. In line with this, the Inter-Agency Coordination Group (IACG) on AMR is currently working to provide guides for approaches to ensuring sustained and effective actions against AMR. In particular, this group recognized the key role of research and development by establishing a specific working group on this issue [7].

Drivers that support mechanisms for the spread of AMR include the growing and often inappropriate use of antimicrobials, in both the medical and veterinary fields. One example is the use of antimicrobials, either prophylactically or therapeutically, for viral infections [2]. The traditional approach to combat AMR includes protocols to promote antimicrobial stewardship, surveillance, prevention and control of infections, research for the development of new antimicrobial molecules, and the restriction of antimicrobial use in veterinary field [8,9,10,11,12,13,14]. However, the research into new molecules is not proving easy to follow for two main reasons: at least ten years are needed to get from the research to marketing stage of an antimicrobial, and the time that elapses between the use of a new antimicrobial and the development of mechanisms of resistance has been increasingly shortened over the years [2,15]. 

In this complex scenario, there are several well-documented and epidemiologically supported examples of both bacterial and viral vaccines having an impact on AMR [16,17]. In particular, some vaccination strategies have been intentionally used to combat the spread of a multidrug-resistant pathogens [18]. Important examples of currently licensed vaccines employed in the prevention of AMR include conjugate vaccines against Haemophilus Influenzae Type B and Pneumococcus. Moreover, future vaccines under development include those for Group B Streptococcus and Staphylococci [18]. In addition to this direct effect on the target pathogens, viral vaccines could indirectly reduce the use of antimicrobials and, therefore, the emergence of AMR [16,17,19]. Accordingly, some authors suggest that a reduction in AMR rates may be more easily achieved in many countries through vaccination policies rather than other interventions, such as hygiene and sanitation [20]. 

Vaccination is certainly the most effective measure against influenza, and, at the European level, the strategy is generally to protect people at higher risk of complications if infected. Accordingly, the WHO recommends influenza vaccines for pregnant women, children aged 6–59 months, the elderly, healthcare workers, and individuals with chronic medical conditions. In 2003, the World Health Assembly offered its support to reach the auspicial target of 75% vaccination coverage in older age groups by 2014–2015, a coverage level settled by the Health Council of all Health Ministers. However, despite several efforts, influenza vaccination coverage is lower than this target in almost all European countries [21]. In fact, influenza vaccination coverage largely varies between countries in the European region. Its values range from 1% to over 75% among the elderly, the target group for which most data exist [21]. Low vaccination coverage is mainly caused by limited vaccine procurement in countries where influenza is not considered a high-priority disease. Instead, variations in vaccine uptake might be attributed to other factors in countries where vaccines are more widely available [21]. Some of these factors include lack of confidence in the vaccine, low awareness of the benefits of vaccination, and lack of recommendation from healthcare providers.

Given this, our hypothesis was that influenza vaccines might at least partially mitigate the spread of AMR pathogens at international and national levels. To explore this hypothesis, in the current study, we focused on Italy, one of the Member States with the highest level of AMR in Europe [22]. This is notably due to the strict connection between AMR, community-acquired and healthcare-associated infections [23,24,25,26]. In 2017, a country visit to Italy by the European Centre for Disease Prevention and Control (ECDC) team allowed for a discussion and assessment of the national situation regarding the prevention and control of AMR through the appropriate use of antimicrobials, antibiotics and infection control measures. Within that framework, the ECDC team raised concerns, particularly about resistance to carbapenems and colistin in gram-negative bacteria, such as *K. pneumoniae* and *A. baumannii* [22]. For all these reasons, in the present study, we conducted an ecological analysis of Italian data to evaluate the relationship between influenza vaccination coverage and AMR proportions over the last two decades.

## 2. Materials and Methods

### 2.1. Data Sources

We first collected national data on influenza vaccination coverage in the overall population and among those aged more than 64 years. These data were released by the Italian National Institute of Health (Istituto Superiore di Sanità, ISS) [27] and made available for each influenza season from 1999–2000 to 2020–2021. It is worth mentioning that the Italian Ministry of Health offers influenza vaccines for all individuals at higher risk of complications, due to age over 64 years or clinical and professional conditions. The Trivalent Inactivated influenza Vaccines (TIVs) were the only vaccines used in Italy until 2014–2015, to which the Quadrivalent Influenza Vaccine (QIV) was added [28]. Data on the number of vaccines administered are routinely collected through immunization registries implemented at the regional level [29]. Furthermore, since the 2010–2011 influenza season, the ISS created an electronic platform for recording data on the number of doses administered, by age group, target category and type of vaccine [27].

We also collected data on AMR percentages for selected isolates and specific periods in Italy, when available. AMR percentages on the total of tested isolates were provided by the Italian AMR surveillance project of the ISS (AR-ISS), which was based on a network of sentinel laboratories reporting data on the antimicrobial susceptibility of bloodstream isolates of selected species [30]. The AR-ISS is based on a set of local and clinical laboratories and reports data to the European Antimicrobial Resistance Surveillance Network (EARS-Net) of the ECDC. The AR-ISS started in 2001, involving 62 sentinel microbiological laboratories throughout the Italian country [30]. According to the last report published in 2021, this number increased to 153 sentinel laboratories, which corresponded to a national coverage of 47.3% [31]. In the current study, data on the total number of isolates tested and percentages of AMR isolates were obtained from the Surveillance Atlas of Infectious Diseases of the ECDC [32] and were related to the following isolates and antimicrobials:

- *A. baumannii* resistant to aminoglycosides, carbapenems, or fluoroquinolones;
- *Escherichia coli* resistant to fluoroquinolones, third-generation cephalosporins, aminoglycosides, or aminopenicillins;
- *K. pneumoniae* resistant to Carbapenems, third-generation Cephalosporins, Aminoglycosides, or Fluoroquinolones;
- *Staphylococcus aureus* resistant to Methicillin (MRSA);
- *Pseudomonas aeruginosa* resistant to Fluoroquinolones, Piperacillin and Tazobactam, Carbapenems, or Ceftazidime;
- *Streptococcus pneumoniae* resistant to Penicillins or Macrolides.

Additionally, data on antimicrobial use in the general population were extracted from the last National Report on Antibiotic use in Italy, published by the Italian Medicines Agency through the Medicines Utilisation Monitoring Centre in 2020 [33]. Available data referred to the period from 2013 to 2019. Antibiotic use was expressed in terms of standard doses (daily defined doses, DDD) consumed per 1,000 persons each day. In particular, DDD represent the number of doses for each drug that are necessary for one day of adult treatment [33]. 

To consider the potential effect of socio-demographic indicators, data on the annual number of Italian residents, proportion of men, and aging index were provided and obtained by the Italian National Institute of Statistics (Istituto Nazionale di Statistica, ISTAT) [34]. In particular, aging index was calculated as the number of residents aged 65 years and over per 100 residents younger than 14 years old [34].

### 2.2. Statistical Analysis

Summary statistics were used to describe influenza vaccination coverage, AMR percentages and other collected measures, using mean, standard deviation (SD), and range, when necessary. First, we tested for bivariate correlations between influenza vaccination coverage and AMR percentages, using the Spearman’s rank correlation coefficient (rho). We also tested for cross-correlations between the time-series with a maximum lag/lead time of 3 years. For combinations with statistically significant correlations, we plotted the annual influenza vaccination coverage against AMR percentages through overlaid scatter plots. To consider potential confounding factors, we also evaluated the correlations between influenza vaccination coverage and AMR proportions and the number of tested isolates, data on antibiotic use, and socio-demographic indicators. Finally, we performed linear regression analyses, using influenza vaccination coverage as the independent variable and percentage of AMR as the dependent variable. For each combination, the analyses were adjusted for the annual number of tested isolates and the aging index. The results were reported as β and its standard error (SE). All the analyses were performed on the SPSS software (version 23.0, SPSS, Chicago, IL, USA), with a significance level α of 0.05.

## 3. Results

### 3.1. Influenza Vaccination Coverage

**Figure 1:**
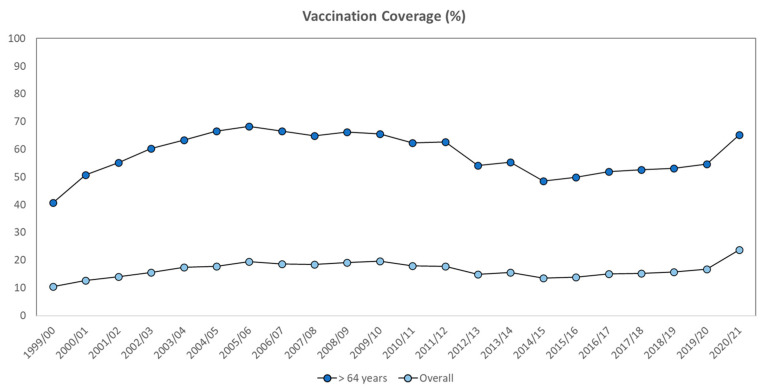
Influenza vaccination coverage in Italy from 2000 to 2021.

Figure 1 shows influenza vaccination coverage in the overall Italian population and among those over 64 years, from 1999–2000 to 2020–2021. In general, the average vaccination coverage was 16.5% in the overall population and 58.2% in the population over 64 years. Vaccination coverage varied over the years without a statistically significant trend. However, for vaccination coverage in the population over 64 years, we observed an increase from 1999–2000 to 2005–2006, a decline from 2006–2007 to 2014–2015, and another increase from 2015–2016 to date. Similar but less marked trends were observed for vaccination coverage in the overall population. As was also reported for population over 64 years, influenza vaccination coverage in the overall population has increased in recent years. 

### 3.2. Antimicrobial Resistance and Antibiotic Use

Table 1 summarizes the percentages of AMR for different isolates and antimicrobials, describing the number of tested isolates, the observed ranges of AMR, and the reference periods for each combination of isolates and antimicrobials. The total number of tested isolates was 44,194 for *E. coli*, 34,455 for *K. pneumoniae*, 8868 for *A. baumannii*, 58,468 for *S. aureus*, 6014 for *S. pneumoniae*, and 17,963 for *P. aeruginosa*. Notably, the annual number of tested isolates increased over the years. The highest average percentages of AMR were reported for *A. baumannii*, independent of the considered antimicrobial. By contrast, the average percentages of AMR varied for *E. coli*: from 15.1% for aminoglycosides to 61.9% for aminopenicillins. A wide variation was also observed for *K. pneumoniae*: from 21.7% for carbapenems to 46.7% for third-generation cephalosporins. The average percentage of *S. aureus* resistant to methicillin was 36.5%, while lower percentages were reported for *P. aeruginosa* and *S. pneumoniae*, respectively. Regarding antibiotic use in the general population, the average value was of 16.9 DDD per 1000 persons each day. The average antibiotic use referred to the period from 2013 to 2019, and its annual value slightly decreased from 18.4 in 2013 to 15.6 in 2019. 

### 3.3. Correlation between Influenza Vaccination Coverage and Antimicrobial Resistance

**Figure 2:**
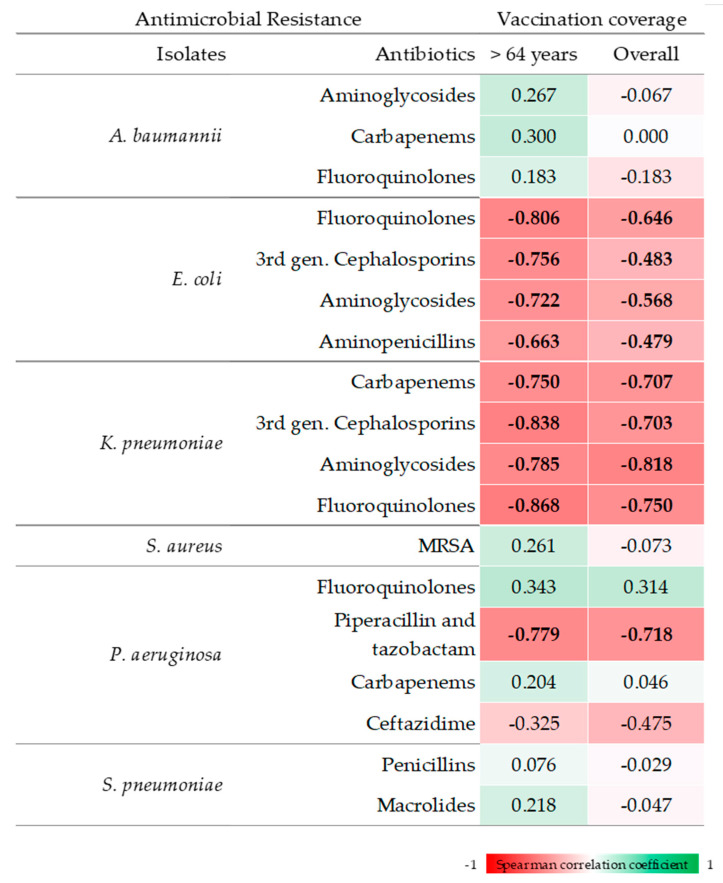
Spearman’s Correlations between influenza vaccination coverage and antimicrobial resistance. Correlations coefficients with *p* < 0.05 are indicated in bold font.

Figure 2 shows Spearman’s correlation coefficients between influenza vaccination coverage and AMR percentages for selected isolates and antimicrobials. We found significant negative correlations between vaccination coverage in the population over 64 years and percentages of AMR of *E. coli* and *K. pneumoniae* (*p*-values < 0.001). Regarding *E. coli*, the greatest correlation coefficient was observed for resistance to fluoroquinolones, followed by third-generation cephalosporins, aminoglycosides, and aminopenicillins. Cross-correlation analysis demonstrated that the highest correlation coefficients were obtained without a time lag between the time-series. The only exception was for *E. coli* that were resistant to aminoglycosides, which showed the highest correlation coefficient at a 2-year lag. Regarding *K. pneumoniae*, the greatest correlation coefficient was observed for fluoroquinolones, followed by third-generation cephalosporins, carbapenems, and aminoglycosides. Cross-correlation analysis demonstrated that the highest correlation coefficients were obtained without a time lag between the time-series. A negative correlation was also observed for *P. aeruginosa* that were resistant to piperacillin and tazobactam (*p* = 0.001), while no significant correlations were evident for the remaining combinations of isolates and antimicrobials. Similar results were obtained when considering influenza vaccination coverage in the over-64 population. In particular, vaccination coverage negatively correlated with percentages of AMR of *E. coli* and *K. pneumoniae* (*p*-values < 0.001). Regarding *E. coli*, the greatest correlation coefficient was observed for resistance to fluoroquinolones, followed by aminoglycosides, third-generation cephalosporins, and aminopenicillins. Regarding *K. pneumoniae*, the greatest correlation coefficient was observed for aminoglycosides, followed by fluoroquinolones, carbapenems, and third-generation cephalosporins. A negative correlation was also observed for *P. aeruginosa* that were resistant to piperacillin and tazobactam (*p* = 0.001), while no significant correlations were evident for the remaining combinations of isolates and antimicrobials.

### 3.4. Correlations of Vaccination Coverage and Antimicrobial Resistance with Socio-Demographic Indicators, Antibiotic Use, and Isolates Tested

To test whether other measures and indicators might confound the observed relationships, we evaluated potential correlations of influenza vaccination coverage with socio-demographic indicators, data on antibiotic use, and number isolates tested each year (Supplementary Table S1). Notably, vaccination coverage in population over 64 years did not correlate with the annual number of Italian residents and the proportion of men (*p*-values > 0.1). With respect to the aging index, we observed a negative but non-significant correlation (rho = −0.495; *p* = 0.072). Similarly, vaccination coverage did not correlate with antibiotic use (*p* = 0.589), although this analysis was limited to the 2013–2019 period. Finally, we found negative correlations with the number of *K. pneumoniae* and *P. aeruginosa* tested (rho = −0.650; *p* = 0.006 and rho = −0.644; *p* = 0.007, respectively), but not with other isolates (*p*-values > 0.05). No correlations were evident between vaccination coverage in the overall population and the abovementioned measures and indicators. 

We also tested for correlations between AMR proportions, socio-demographic indicators, data on antibiotic use, and the number of isolates tested each year. The analysis was limited to specific combination of isolates and antimicrobials exhibiting significant correlations with vaccination coverage (i.e., *E. coli* resistant to fluoroquinolones, third-generation cephalosporins, aminoglycosides, and aminopenicillins; *K. pneumoniae* resistant to fluoroquinolones, third-generation cephalosporins, carbapenems, and aminoglycosides; *P. aeruginosa* resistant to piperacillin and tazobactam) (Supplementary Table S2). Notably, AMR proportions did not correlate with the annual number of Italian residents, the proportion of men, and antibiotic use (*p*-values > 0.1). By contrast, the aging index showed positive correlations with the proportion of *E. coli* resistant to third generation cephalosporins (rho = 0.855; *p* < 0.001) and aminopenicillins (rho = 0.534; *p* = 0.049), with the proportion of *K. pneumoniae* resistant to fluoroquinolones (rho = 0.736; *p* = 0.003), third-generation cephalosporins (rho = 0.749; *p* = 0.002) and carbapenems (rho = 0.604; *p* = 0.022), and with *P. aeruginosa* resistant to piperacillin and tazobactam (rho = 0.560; *p* = 0.037). Moreover, we observed positive correlations between AMR proportions and the number of isolates tested for all microorganisms under investigation (*p*-values < 0.05). 

### 3.5. Linear Relationships between Vaccination Coverage and Antimicrobial Resistance

**Figure 3:**
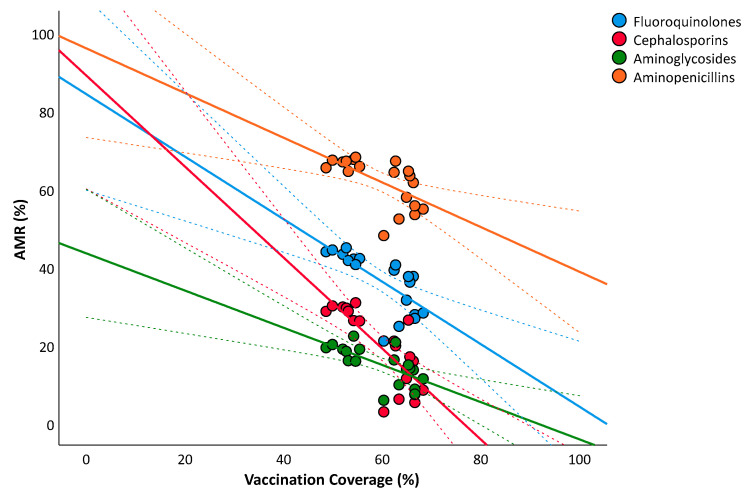
The relationship between influenza vaccination coverage in population over 64 years and antimicrobial resistance of *Escherichia coli*. The plot shows linear regression lines and their 95%CI (dotted lines).

Next, we tested the linear relationship between influenza vaccination coverage in population over 64 years and AMR of *E. coli* and *K. pneumoniae*. Figure 3 represents the overlaid scatter plot of the relationship between influenza vaccination coverage and percentages of *E. coli* resistant to fluoroquinolones, third-generation cephalosporins, aminoglycosides, and aminopenicillins. After adjusting for the number of tested isolates and for the aging index, the analysis confirmed significant and negative relationships with percentages of resistance to third-generation cephalosporins (β = −0.639; SE = 0.101; *p* < 0.001), fluoroquinolones (β = −0.487; SE = 0.107; *p* = 0.001), aminoglycosides (β = −0.361; SE = 0.105; *p* = 0.006), and aminopenicillins (β = −0.267; SE = 0.111; *p* = 0.037). 

**Figure 4:**
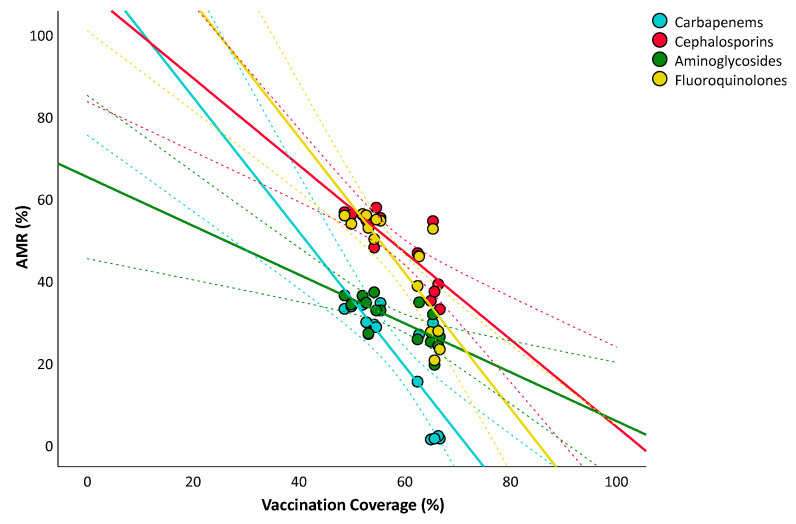
The relationship between influenza vaccination coverage in population over 64 years and antimicrobial resistance of *Klebsiella pneumoniae*. The plot shows linear regression lines and their 95% CI (dotted lines).

Similarly, Figure 4 represents the overlaid scatter plot of the relationship between influenza vaccination coverage and percentages of *K. pneumoniae* resistant to carbapenems, third-generation cephalosporins, aminoglycosides, and fluoroquinolones. After adjusting for the number of tested isolates and for the aging index, the analysis confirmed significant and negative relationships with percentages of resistance to carbapenems (β = −1.241; SE = 0.513; *p* = 0.036), fluoroquinolones (β = −1.207; SE = 0.413; *p* = 0.015), and third-generation cephalosporins (β = −0.735; SE = 0.226; *p* = 0.009), but not with aminoglycosides (β = −0.465; SE = 0.284; *p* = 0.133). A negative relationship was also evident for *P. aeruginosa* that were resistant to piperacillin and tazobactam (β = −0.609; SE = 0.229; *p* = 0.024).

We also tested the linear relationship between influenza vaccination coverage in the overall population and AMR of *E. coli* and *K. pneumoniae*. Regarding *E. coli*, the analysis confirmed a significant and negative relationship with the percentages of resistance to aminoglycosides (β = −0.789; SE = 0.251; *p* = 0.010), third-generation cephalosporins (β = −1.401; SE = 0.262; *p* < 0.001), fluoroquinolones (β = −1.085; SE = 0.254; *p* = 0.002), and aminopenicillins (β = −0.608; SE = 0.253; *p* = 0.037), after adjusting for covariates. 

Regarding *K. pneumoniae*, the analysis confirmed significant and negative relationships with the percentages of resistance to fluoroquinolones (β = −2.378; SE = 0.934; *p* = 0.029) and third-generation cephalosporins (β = −1.396; SE = 0.531; *p* = 0.025), after adjusting for covariates. By contrast, no significant relationship was evident for resistance to aminoglycosides (β = −0.860; SE = 0.421; *p* = 0.199) and carbapenems (β = −2.266; SE = 1.176; *p* = 0.083).

## 4. Discussion

To our knowledge, the present study is the first to evaluate the relationship between influenza vaccination coverage and AMR at population level. To do that, we performed an ecological analysis of Italian data, which revealed an inverse correlation between influenza vaccination coverage and AMR for selected microorganisms and antimicrobials. 

These findings, however, should be interpreted with caution due to the potential effect of unmeasured and non-available factors that might influence and/or mediate the observed relationship. In fact, several other factors contribute to AMR besides influenza vaccination, such as socio-demographic and patient’s characteristics, health conditions, vaccine type and supply, local public health condition, medication, incidence of infections, and antibiotic use. In our study, we also evaluated the relationships of influenza vaccination coverage with some socio-demographic indicators (i.e., number of residents, proportion of men, and aging index), the number of isolates tested annually, and antibiotic use in the general population. Accordingly, we noted an inverse but non-significant correlation with the aging index of the Italian population, and a significant inverse correlation with the number of tested isolates (i.e., *K. pneumoniae* and *P. aeruginosa*). Antibiotic use in the general population, instead, did not correlate with influenza vaccination coverage. Based on these results, we also performed linear regression analyses by adjusting for the aging index and for the number of isolates that were tested annually. In particular, influenza vaccination coverage in the population over 64 years exhibited an inverse relationship with the proportions of AMR in *E. coli* and *K. pneumoniae*. For *E. coli*, inverse relationships were evident for the percentages of resistance to third-generation cephalosporins, fluoroquinolones, aminoglycosides, and aminopenicillins. For *K. pneumoniae*, inverse relationships were evident for the percentages of resistance to carbapenems, fluoroquinolones, and third-generation cephalosporins. A negative relationship was also evident for *P. aeruginosa* that was resistant to piperacillin and tazobactam. Similar results were obtained considering vaccination coverage in the overall population. 

In general, these findings suggested that the proportion of antimicrobial-resistant microorganisms decreased with increasing vaccination coverage over the last two decades. However, the mechanisms underpinning this potential relationship should be better investigated and then clarified. Although no formal studies have been conducted to investigate if any immunological mechanisms could support this evidence, it is plausible to assume that influenza vaccination coverage might act through indirect mechanisms [16]. In fact, there are two supposed ways by which influenza vaccination might reduce AMR spread: it prevents secondary bacterial infections and reduces the prescription of antimicrobials. Beyond preventing influenza infections and disease, influenza vaccines can also reduce the risk of pneumonia and otitis media [35]. For instance, a study conducted on Turkish children during the influenza season demonstrated a significant reduction in the incidence of otitis media among those receiving the influenza vaccine [36]. This also had a ripple effect on antimicrobial use, so fewer antibiotic prescriptions were reported in children receiving the influenza vaccine [36]. A similar effect also occurred with acute febrile illnesses, which are often treated inappropriately with antimicrobials. Another benefit of influenza vaccination against AMR spread is the reduction in inappropriate antimicrobial prescriptions to treat viral respiratory tract infections [17]. In fact, it has been demonstrated that nearly half of antimicrobials are inappropriately prescribed for respiratory tract infections associated with pathogens that are not susceptible to antimicrobials [37]. A Canadian study showed a significant reduction in antimicrobial prescriptions among children after the introduction of universal influenza vaccination in Ontario [38]. Similarly, a multicenter trial in Europe demonstrated a lower incidence of influenza and a reduction in antimicrobial use in children receiving the influenza vaccine than in those randomized to the placebo group [39]. Another study in England, investigating the impact of the influenza vaccination program on antibiotic prescribing rates, found no evidence of a reduction in prescriptions for respiratory tract infections but a significant inverse relationship with prescriptions at the general practice level [40]. In our study, no relationship was evident between influenza vaccination coverage and antibiotic use in the general population. However, it is worth mentioning that antibiotic use varied slightly over the years, and that such data were limited to 2013–2019. Although some previous findings support the hypothesis of an indirect effect of influenza vaccination on reducing AMR, this did not explain why the inverse relationship was evident only for *E. coli* and *K. pneumoniae*. One reason for this may lie in the percentages of resistance of these pathogens, which were extremely varied over the years. Instead, lower variations in AMR percentages were observed for other pathogens, which probably prevented a significant association with vaccination coverage. It is worth underlining that this issue also regarded influenza vaccination coverage, which only ranged from 40% to 68%. 

Despite the general consensus that influenza vaccination might be beneficial against AMR, measuring its potential impact is challenging because the data are still fragmented and difficult to retrieve. To address this issue, the Global Influenza Initiative (GII) Work Stream II Team conducted a mixed-methods study, which included a literature review of research articles, public health reports, and national action plans against AMR and ad-hoc interviews with experts on influenza and AMR [41]. Regarding research articles, the team identified 18 randomized controlled trials and 16 observational studies, reporting how influenza vaccination might reduce the number of antibiotic courses and the number of people receiving antibiotics. From a total of 18 public health reports, eight specifically discussed the relationship between influenza vaccination and AMR. Finally, the review of national action plans identified seven reports suggesting influenza vaccination as a useful approach against AMR. Interviews with twelve policy and public experts raised several points for discussion: (i) policy makers and physicians did not consider AMR as a primary argument for vaccination; (ii) influenza vaccination should be a crucial point in all AMR national action plans, although these plans are often not really implemented; (iii) there is a general need for more efforts to increase influenza vaccination coverage, especially considering the potential impact of the COVID-19 pandemic [41]. Following a review of the literature and expert interviews, the GII team drew up a set of recommendations. From a policy perspective, it is necessary to increase influenza vaccination coverage with targeted programs and to include influenza vaccination in AMR national action plans. Accordingly, all plans should be reviewed, with a particular focus on their implementation, information and recommendations. From a scientific perspective, it is necessary to properly define the effects of influenza vaccination on AMR by increasing the quality of data on this relationship. To do this, more funding is needed, even to sustain the research and development of new antimicrobials and universal influenza vaccines [41]. 

As stated above, our results should be interpreted cautiously due to the ecological nature of our analysis [42] and the several weaknesses that characterized it. This kind of study, in fact, does not examine individuals but populations, and the results are, therefore, prone to biases and misinterpretations. Thus, the observed relationships do not mean that influenza vaccination exerts a causal effect on AMR. Instead, they generate hypotheses for further investigation. 

Data coverage was one of the main limitations of our study. Data related to vaccination coverage indicate the number of vaccine doses administered over the overall population or residents aged over 64 years. However, certain inaccuracies cannot be completely excluded, mainly due to the decentralized Italian Health System and to the different characteristics and functionalities of immunization registries implemented at regional levels [29]. Despite this great progress, we are still far from reaching an optimal situation in Italy. Different software solutions are used, sometimes within the same region, which complicates the aggregation of data, which are often collected in multiple ways [29]. Thus, to make the collection and estimation of vaccination coverage more accurate, future efforts will have to focus on interoperability among the systems adopted at the various levels. A similar issue pertained to data on AMR, which were provided by a network of sentinel laboratories operating in the national territory. Although the number of sentinel laboratories increased from 2001 to 2021, the national coverage is still low (i.e., nearly 50%) [31]. For these reasons, measures of exposure and outcome were only proxies of the real situation in Italy. Moreover, these measures were based on the average value in the population, impeding generalizations at the individual level. These measures, collected in different ways that were not integrated with each other, referred to different samples from the Italian population. Another limitation of our study regarded the general lack of data on confounding and mediating factors. This did not allow for consideration of, or adjustment for, other factors that influence the outcome [42]. However, additional factors should be considered, including vaccine supply, socio-cultural characteristics, and health conditions at individual and community level. A stratified analysis would have shown differences by type of influenza vaccine. Moreover, it would have been appropriate to have reliable information at the population level on antibiotic use, incidence of infections sustained by AMR pathogens, medications and hospitalizations due to these infections. This would have made it possible to define a stronger and clearer picture of the relationship between influenza vaccination coverage and AMR. 

## 5. Conclusions

In conclusion, the present study proposed an inverse relationship between influenza vaccination coverage and AMR. Our results were in accordance with previous theories illustrating how influenza vaccination reduced prescriptions for secondary bacterial infections, as well as the inappropriate antimicrobial use against acute febrile illnesses. However, our study design and results did not allow us to demonstrate this effect. For this reason, more and better data on confounding and mediating factors should be available to confirm our findings and to elucidate the mechanisms underpinning this relationship. The positive outlook is that future vaccines targeting other respiratory viruses could result in an even more marked reduction in antimicrobial use and AMR. For these reasons, further research should be encouraged to better evaluate the effectiveness of viral vaccination programs in impacting AMR.

## Figures and Tables

**Table 1:** Antimicrobial resistance in Italy for specific isolates and antibiotics.

Species	Annual Number of Isolates Tested	Antimicrobials	Period	% Resistance		
				Mean	SD	Range
*A. baumannii*	236–2522	Aminoglycosides	2012–2020	79.6	4.3	74.7–88.3
		Carbapenems	2012–2020	80.8	3.7	78.3–89.9
		Fluoroquinolones	2012–2020	83.2	3.9	79.2–92.1
*E. coli*	564–7533	Fluoroquinolones	2002–2020	36.6	7.5	21.1–44.9
		3rd gen. Cephalosporins	2002–2020	19.6	9.9	2.9–30.9
		Aminoglycosides	2002–2020	15.1	4.7	5.9–22.3
		Aminopenicillins	2002–2020	61.9	6.2	48.0–68.1
*K. pneumoniae*	305–8293	Carbapenems	2006–2020	21.7	13.5	1.1–34.3
		3rd gen. Cephalosporins	2005–2020	46.7	11.1	19.5–57.6
		Aminoglycosides	2005–2020	28.9	7.7	7.9–37.0
		Fluoroquinolones	2005–2020	42.4	15.3	11.3–56.1
*S. aureus*	470–10923	Methicillin	2000–2020	36.5	3.0	33.5–44.3
*P. aeruginosa*	151–4537	Fluoroquinolones	2006–2020	28.9	6.3	19.6–42.0
		Piperacillin and tazobactam	2006–2020	23.1	5.4	13.3–30.6
		Carbapenems	2006–2020	22.8	5.3	13.7–32.9
		Ceftazidime	2006–2020	21.1	3.2	16.2–25.5
*S. pneumoniae*	141–1017	Penicillins	2005–2020	4.1	1.9	0.8–8.6
		Macrolides	2005–2020	25.2	3.8	19.4–33.8

## Data Availability

Data that support the findings of this study are available from the corresponding author, upon reasonable request.

## Supplementary Materials

The following are available online at https://www.mdpi.com/article/10.3390/vaccines10040554/s1, Table S1. Spearman’s Correlations between influenza vaccination coverage, socio-demographic indicators, antibiotic use, and the number of isolates tested; Table S2. Spearman’s Correlations between antimicrobial resistance proportions, socio-demographic indicators, antibiotic use, and the number of isolates tested.

## References

[1] Cassini A., Högberg L.D., Plachouras D., Quattrocchi A., Hoxha A., Simonsen G.S., Colomb-Cotinat M., Kretzschmar M.E., Devleesschauwer B., Cecchini M. (2019). Attributable deaths and disability-adjusted life-years caused by infections with antibiotic-resistant bacteria in the EU and the European Economic Area in 2015: A population-level modelling analysis. Lancet Infect. Dis..

[2] Jansen K.U., Knirsch C., Anderson A.S. (2018). The role of vaccines in preventing bacterial antimicrobial resistance. Nat. Med..

[3] European Centre for Disease Prevention and Control Antimicrobial Resistance Surveillance in Europe 2022–2020 Data. https://www.ecdc.europa.eu/en/publications-data/antimicrobial-resistance-surveillance-europe-2022-2020-data.

[4] Antimicrobial Resistance Collaborators (2022). Global burden of bacterial antimicrobial resistance in 2019: A systematic analysis. Lancet.

[5] World Health Organization Ten Threats to Global Health in 2019. https://www.who.int/news-room/spotlight/ten-threats-to-global-health-in-2019.

[6] World Health Organization One Health Global Leaders Group on Antimicrobial Resistance. https://www.who.int/news-room/articles-detail/one-health-global-leaders-group-on-antimicrobial-resistance.

[7] World Health Organization Antimicrobial Resistance: Invest in Innovation and Research, and Boost R&D and Access 2018. https://www.who.int/antimicrobial-resistance/interagency-coordination-group/IACG_AMR_Invest_innovation_research_boost_RD_and_access_110618.pdf.

[8] Barchitta M., Quattrocchi A., Maugeri A., La Rosa M.C., La Mastra C., Sessa L., Cananzi P., Murolo G., Oteri A., Basile G. (2019). Antibiotic Consumption and Resistance during a 3-Year Period in Sicily, Southern Italy. Int. J. Environ. Res. Public Health.

[9] Newitt S., Oloyede O., Puleston R., Hopkins S., Ashiru-Oredope D. (2019). Demographic, Knowledge and Impact Analysis of 57,627 Antibiotic Guardians Who Have Pledged to Contribute to Tackling Antimicrobial Resistance. Antibiotic.

[10] Chaintarli K., Ingle S.M., Bhattacharya A., Ashiru-Oredope D., Oliver I., Gobin M. (2016). Impact of a United Kingdom-wide campaign to tackle antimicrobial resistance on self-reported knowledge and behaviour change. BMC Public Health.

[11] McCullough A.R., Parekh S., Rathbone J., Del Mar C.B., Hoffmann T.C. (2016). A systematic review of the public’s knowledge and beliefs about antibiotic resistance. J. Antimicrob. Chemother..

[12] Barchitta M., Quattrocchi A., Maugeri A., Rosa M.C., Mastra C., Basile G., Giuffrida G., Rinaldi F.M., Murolo G., Agodi A. (2020). The “Obiettivo Antibiotico” Campaign on Prudent Use of Antibiotics in Sicily, Italy: The Pilot Phase. Int. J. Env. Res. Public Health.

[13] Barchitta M., Sabbatucci M., Furiozzi F., Iannazzo S., Maugeri A., Maraglino F., Prato R., Agodi A., Pantosti A. (2021). Knowledge, attitudes and behaviors on antibiotic use and resistance among healthcare workers in Italy, 2019: Investigation by a clustering method. Antimicrob. Resist. Infect. Control.

[14] Brusaferro S., Arnoldo L., Finzi G., Mura I., Auxilia F., Pasquarella C., Agodi A. (2018). Hospital Hygiene and Infection Prevention and Control in Italy: State of the art and perspectives. Ann. Ig..

[15] Alghamdi S. (2021). The role of vaccines in combating antimicrobial resistance (AMR) bacteria. Saudi. J. Biol. Sci..

[16] Micoli F., Bagnoli F., Rappuoli R., Serruto D. (2021). The role of vaccines in combatting antimicrobial resistance. Nat. Rev. Microbiol..

[17] Klugman K.P., Black S. (2018). Impact of existing vaccines in reducing antibiotic resistance: Primary and secondary effects. Proc. Natl. Acad. Sci. USA.

[18] Jansen K.U., Gruber W.C., Simon R., Wassil J., Anderson A.S. (2021). The impact of human vaccines on bacterial antimicrobial resistance. A review. Env. Chem. Lett..

[19] Esposito S., Principi N. (2018). Influenza vaccination and prevention of antimicrobial resistance. Expert Rev. Vaccines.

[20] Ginsburg A.S., Klugman K.P. (2017). Vaccination to reduce antimicrobial resistance. Lancet Glob. Health.

[21] World Health Organization Influenza Vaccination Coverage and Effectiveness. https://www.euro.who.int/en/health-topics/communicable-diseases/influenza/vaccination/influenza-vaccination-coverage-and-effectiveness.

[22] European Centre for Disease Prevention and Control ECDC Country Visit to Italy to Discuss Antimicrobial Resistance Issues. https://www.ecdc.europa.eu/sites/default/files/documents/AMR-country-visit-Italy.pdf.

[23] Barchitta M., Maugeri A., La Rosa M.C., La Mastra C., Murolo G., Corrao G., Agodi A. (2021). Burden of Healthcare-Associated Infections in Sicily, Italy: Estimates from the Regional Point Prevalence Surveys 2016–2018. Antibiotics.

[24] Barchitta M., Maugeri A., La Rosa M.C., La Mastra C., Murolo G., Agodi A. (2020). Three-Year Trends of Healthcare-Associated Infections and Antibiotic Use in Acute Care Hospitals: Findings from 2016–2018 Point Prevalence Surveys in Sicily, Italy. Antibiotics.

[25] Bordino V., Vicentini C., D’Ambrosio A., Quattrocolo F., Zotti C.M. (2020). Burden of Healthcare-Associated Infections in Italy: Disability-Adjusted Life Years. Eur. J. Public Health.

[26] (2018). Secondo Studio di Prevalenza Italiano sulle Infezioni Correlate All’assistenza e sull’uso di Antibiotici Negli Ospedali per Acuti–Protocollo ECDC.

[27] Italian National Institute of Health Coperture Della Vaccinazione Antinfluenzale in ITALIA. https://www.epicentro.iss.it/influenza/coperture-vaccinali.

[28] Italian Ministry of Health Influenza Vaccination. https://www.salute.gov.it/portale/influenza/dettaglioContenutiInfluenza.jsp?id=686&area=influenza&menu=vuoto&tab=1.

[29] D’Ancona F., Gianfredi V., Riccardo F., Iannazzo S. (2018). Immunisation Registries at regional level in Italy and the roadmap for a future Italian National Registry. Ann. Ig..

[30] Boccia D., D’Ancona F., Salmaso S., Monaco M., Del Grosso M., D’Ambrosio F., Giannitelli S., Lana S., Fokas S., Pantosti A. (2005). Antibiotic-resistance in Italy: Activity of the first year of the surveillance project AR-ISS. Ann. Ig..

[31] Italian National Institute of Health AR-ISS: Sorveglianza Nazionale dell’Antibiotico-Resistenza. https://www.epicentro.iss.it/antibiotico-resistenza/ar-iss/RIS-1_2021.pdf.

[32] European Centre for Disease Prevention and Control Surveillance Atlas of Infectious Diseases. https://atlas.ecdc.europa.eu/public/index.aspx.

[33] Italian Medicines Agency (2019). L’uso Degli Antibiotici in Italia. Rapporto Nazionale. https://www.aifa.gov.it/documents/20142/1283180/Rapporto_Antibiotici_2019.pdf.

[34] Italian National Institute of Statistics. https://www.istat.it/en/.

[35] Kash J.C., Taubenberger J.K. (2015). The role of viral, host, and secondary bacterial factors in influenza pathogenesis. Am. J. Pathol..

[36] Ozgur S.K., Beyazova U., Kemaloglu Y.K., Maral I., Sahin F., Camurdan A.D., Kizil Y., Dinc E., Tuzun H. (2006). Effectiveness of inactivated influenza vaccine for prevention of otitis media in children. Pediatr. Infect. Dis. J..

[37] Fleming-Dutra K.E., Hersh A.L., Shapiro D.J., Bartoces M., Enns E.A., File T.M., Finkelstein J.A., Gerber J.S., Hyun D.Y., Linder J.A. (2016). Prevalence of Inappropriate Antibiotic Prescriptions Among US Ambulatory Care Visits, 2010–2011. JAMA.

[38] Kwong J.C., Maaten S., Upshur R.E., Patrick D.M., Marra F. (2009). The effect of universal influenza immunization on antibiotic prescriptions: An ecological study. Clin. Infect. Dis..

[39] Dbaibo G. Inactivated quadrivalent influenza vaccine reduces influenza associated healthcare, antibiotic use and parent-child absenteeism during a randomized controlled trial in healthy children aged 6–35 months. Proceedings of the 35th Meeting of the European Society for Paediatric Infectious Diseases.

[40] Muller-Pebody B., Sinnathamby M.A., Warburton F., Rooney G., Andrews N., Whitaker H., Henderson K.L., Tsang C., Hopkins S., Pebody R.G. (2021). Impact of the childhood influenza vaccine programme on antibiotic prescribing rates in primary care in England. Vaccine.

[41] Global Influenza Initiative Work Stream II Team Influenza Vaccination and Antimicrobial Resistance: Strategic Recommendations. https://www.nivel.nl/sites/default/files/bestanden/1003995.pdf39.

[42] Sedgwick P. (2014). Ecological studies: Advantages and disadvantages. BMJ Br. Med. J..

