# Supplementary material for: The Inverse Relationship between Influenza Vaccination and Antimicrobial Resistance: An Ecological Analysis of Italian Data
Source: Vaccines (Basel). 2022 Apr 3;10(4):554. doi: 10.3390/vaccines10040554 (PMC9030332; doi:10.3390/vaccines10040554)
Supplement: Supplementary file 1 [file vaccines-10-00554-s001.zip › vaccines-1612981-supplementary.pdf]

SUPPLEMENTARY FILE

Supplementary Table S1. Spearman's Correlations between influenza vaccination coverage, socio-demographic indicators, antibiotic use, and the number of isolates tested. Correlations coefficients with $p < 0.05$ are indicated in bold font.

**Correlations**	**Vaccination coverage**	
	**Overall**	**> 64 years**
**Socio-demographic indicators**		
Annual number of residents	0.017	0.025
Proportion of men	0.234	0.145
Aging Index	−0.495	−0.279
**Antibiotic Use**	−0.250	−0.643
**Number of isolates tested**		
A. baumannii	0.433	**0.833**
E. coli	−0.373	−0.016
K. pneumoniae	**−0.650**	−0.421
S. aureus	−0.390	−0.104
P. aeruginosa	**−0.644**	−0.388
S. pneumoniae	−0.235	−0.015

Supplementary Table S2. Spearman's Correlations between antimicrobial resistance proportions, socio-demographic indicators, antibiotic use, and the number of isolates tested. Correlations coefficients with $p < 0.05$ are indicated in bold font.

**Correlations**	**E. coli**				**K. pneumoniae**				**P. aeruginosa**
	**Fluoroquinolones**	**3rd gen. cephalosporins**	**Aminoglycosides**	**Aminopenicillins**	**Fluoroquinolones**	**3rd gen. cephalosporins**	**Carbapenems**	**Aminoglycosides**	**Piperacillin and tazobactam**
**Socio-demographic indicators**									
Annual number of residents	0.384	0.134	0.222	0.118	0.400	0.388	0.376	-0.011	0.135
Proportion of men	0.124	0.257	0.301	0.344	0.234	0.255	0.111	0.145	0.265
Aging Index	0.473	**0.855**	0.187	**0.543**	**0.736**	**0.749**	**0.604**	0.393	**0.560**
**Antibiotic Use**	0.393	−0.643	0.750	−0.321	0.036	0.036	0.521	0.286	0.679
**Number of isolates tested**									
E. coli	**0.794**	**0.902**	**0.619**	**0.737**	-	-	-	-	-
K. pneumoniae	**-**	-	-	-	**0.821**	**0.815**	**0.664**	**0.547**	-
P. aeruginosa	**-**	-	-	-	-	-	-	-	**0.632**
